# Responses to novelty in wild insular birds: comparing breeding populations in ecologically contrasting habitats

**DOI:** 10.1007/s10071-024-01838-w

**Published:** 2024-03-02

**Authors:** Samara Danel, Nancy Rebout, Léna Bureau, Timothée Zidat, Dora Biro, Francesco Bonadonna

**Affiliations:** 1 Department of Brain and Cognitive Sciences, University of Rochester, Rochester, NY 14627 USA; 2 Université Clermont Auvergne, INRAE, VetAgro Sup, UMR Herbivores, 63122 Saint-Genès-Champanelle, France; 3 CEFE, Univ Montpellier, CNRS, EPHE, IRD, Montpellier, France; 4 School of Biosciences, Cardiff University, Museum Avenue, Cardiff, Wales CF10 3AX UK

**Keywords:** Charadriiformes, Exploration, Field experiment, Island, Novelty, Sheathbills

## Abstract

Islands have always provided ideal natural laboratories for assessing ecological parameters influencing behaviour. One hypothesis that lends itself well to testing in island habitats suggests that animals frequenting highly variable environments should be motivated to approach and interact with (i.e. explore) novelty. Intra-species comparisons of populations living in ecologically different island habitats may, thus, help reveal the factors that modulate animals’ responses to novelty. In this study, we presented novel objects to two geographically isolated breeding populations of the black-faced sheathbill (*Chionis minor*), a sedentary land-based bird that frequents remote sub-Antarctic islands. In the first population (*Chionis minor* ssp. *crozettensis*), the “Crozet group” (*Baie du Marin*, Ile de la Possession, Crozet Islands), breeding pairs inhabit a variable habitat close to penguin (*Aptenodytes patagonicus*) colonies. In the second population (*Chionis minor* ssp. *minor*), the “Kerguelen group” (*île Verte*, Morbihan gulf, Kerguelen Islands) breeding pairs live in penguin-free territories. In this latter population, the environment is less variable due to the presence of a broad intertidal zone which ensures year-round food availability. At both Kerguelen and Crozet, at least one breeding partner in all pairs approached at least one of the novel objects, and we found no significant differences in the latency of approach between the two populations. However, sheathbills at Crozet touched objects significantly more than birds at Kerguelen, and were also faster to touch them. We discuss how environmental variability, along with other potential influencing factors, may favour exploration of novelty in this wild insular bird.

**Supplementary Information:**

The online version contains supplementary material available at 10.1007/s10071-024-01838-w.

## Introduction

Responses to novelty have been extensively studied in animals, with birds in both urban and non-urban contexts being a particular focus for research (e.g. Biondi et al. 2020; Huang et al. 2020; Inzani et al. 2023). Yet, comparative analyses of responses to novelty have revealed mixed patterns of findings (Griffin et al. 2017). Since ecological parameters can vary considerably from one island to another, remote archipelagos of the Southern Indian Ocean provide useful, complementary sites for empirically testing how responses to novelty relate to environmental variation. For instance, interacting with novel objects to gain information, here defined as exploration (O’Hara et al. 2017), may facilitate exploitation of novel resources and should be exhibited in populations living in highly variable habitats where exploration can be valuable in identifying novel resources in times of scarcity (Greenberg and Mettke-Hofmann 2001). In parallel, assessing novelty exploration in animals can help developing relevant conservation applications. For instance, in a recent comparative study, hatchings of two crocodilian species were exposed to a novel object (Reber et al. 2021). One species (American alligators, *Alligator mississippiensis*) was more explorative than the other (spectacled caimans, *Caiman crocodilus*), and this was attributed to the efficiency of their parental protection and the resulting decrease in predator avoidance. Such knowledge regarding species-specific behavioural exploration may, in turn, inform efforts to reintroduce captive-bred juveniles to the wild, e.g. by extending the raising period in captivity to increase body size or by releasing less explorative individuals.

In this study, we aimed to investigate whether exploration varies in two subspecies of a sedentary and territorial breeding bird, the black-faced sheathbill (*Chionis minor,* hereafter: sheathbill). Sheathbills have few predators (e.g. giant petrels, *Macronectes giganteus*, skuas, *Catharacta antarctica* ssp. *lonnbergi*), are omnivorous, and show high opportunism and flexible foraging behaviour (Burger and Kirwan 2020). In the “Crozet group” (*Chionis minor* ssp. *crozettensis*; *Baie du Marin*, Crozet Islands), sheathbills breed near a colony of king penguins (*Aptenodytes patagonicus*) in a highly unpredictable environment where food is scarce during winter (Verheyden and Jouventin 1991). At this location, sheathbills feed on deserted penguin eggs, chicks, faeces of seals and seabirds, carrion, blood, seal placenta, and invertebrates (e.g. insects). This species is also known to kleptoparasitize penguins when they regurgitate food (e.g. krill, fish, squid) for their chick. By contrast, the extensive intertidal zone allows birds from the “Kerguelen group” (*Chionis minor* ssp. *minor*; *île Verte,* Kerguelen Islands) to live in a more predictable environment (Jouventin et al. 1996). At this site, sheathbills feed on algae, invertebrates (e.g. mussels, taken at low tide) and petrel carcasses.

We, thus, field tested whether sheathbill breeding pairs (i) *approached* novel objects, resulting from the interaction between attraction (neophilia) and avoidance (neophobia) of novel stimuli, and (ii) *touched* novel objects, reflecting a propensity for exploration (in the absence of external rewards (e.g. Miller et al. 2015)). We predicted that low neophobia, resulting from reduced predation pressure related to insular environments, should favour approach behaviours in both groups (prediction 1). We also expected that high environmental variability should elicit faster approaches (prediction 2), more explorations (prediction 3), and faster explorations (prediction 4), in sheathbills from the Crozet group.

## Methods

### Study subspecies

Twelve wild established breeding pairs of black-faced sheathbills (*Chionis minor*), which frequent different oceanic islands in the sub-Antarctic bio-geographical province (French Southern and Antarctic Lands), participated in this experiment during Nov–Dec 2021. Six breeding pairs of the subspecies *crozettensis* were tested at *Baie du Marin*, Ile de la Possession, Crozet Islands (46°S, 51°E), between 01/11/2021 and 07/11/2021. Six breeding pairs of the subspecies *minor* were located at *île Verte*, Morbihan gulf, Kerguelen Islands (49°S, 69°E), and were tested between 26/11/2021 and 05/12/2021. These two sub-Antarctic subspecies were selected according to their genetic proximity (they have been suggested to diverge recently: (Viot et al. 1993), and generally show no genetic mixing (Burger and Kirwan 2020)) and the contrast in their habitats (Bried and Jouventin 1998). Indeed, the extensive intertidal zone (and thus low environmental variability) is only present at Kerguelen Islands (Bried and Jouventin 1997). All four subspecies of *Chionis minor* possess poor flying abilities (Verheyden and Jouventin 1991) and generally do not disperse at other islands (Burger 1979).

### Experimental setup and materials

**Fig. 1:**
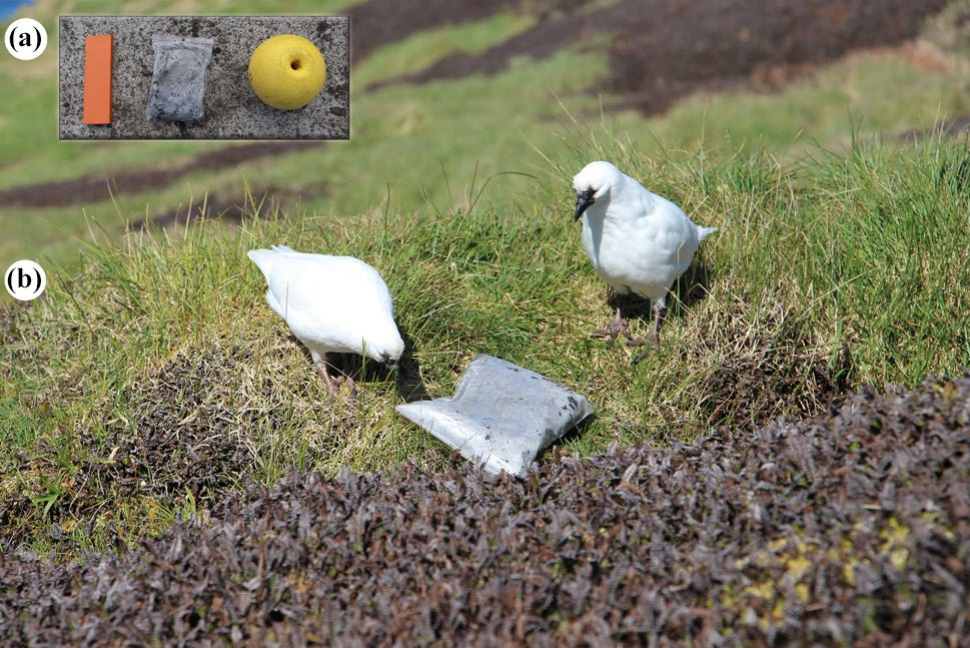
The objects and setup used for assessing novelty responses in two subspecies of black-faced sheathbills (*Chionis minor*). **a** The three novel objects used as stimuli. From left to right: orange wood plank, black plastic bag and yellow buoy, **b** Example of a trial performed by a breeding pair (ssp. *crozettensis*, Crozet group) with the black plastic bag as the novel stimulus

We used three novel objects that varied in colour, shape and size: a yellow buoy (15 cm in diameter, 20 cm in height), a zip lock^®^ plastic bag (15 cm in length, 10 cm in width; filled with black sand to prevent birds flying off with it), and an orange wood plank (25 cm in length, 10 cm in width; Fig. 1a). The camera (GoPro Hero5 black) was placed on the ground at a distance of approximately 8 m from the breeding pair.

### Procedure

When both individuals of the focal breeding pair were simultaneously present on their territory, the experimenter triggered the camera, slowly approached the breeding pair, placed the novel object in front of and at about 1 m distance from the birds, and retreated at least 10 m. Only the breeding partner that first approached the novel objects was included in the analyses (see below). Three trials in total were administered for each breeding pair (1 trial per day for each novel object on 3 successive days, duration of 1 trial: 6 min, timing of trials: 9 a.m. to 4 p.m.), and the order of presentation of the three novel objects was varied randomly across breeding pairs. Each trial started when the experimenter left the test area and ended after the maximum trial duration had elapsed.

### Data analysis

To investigate whether the two groups significantly differed in *approach* (i.e. prediction 1; whether a bird approached to within one body length, including the tail, of the novel object) and *touch* propensities (i.e. prediction 3; whether a bird used its beak to contact the novel object), we built two generalised linear mixed-effects models (GLMM) with a binomial distribution (variable response for (i) prediction 1: coded as 0 = no approach, coded as 1 = approach; (ii) prediction 3: coded as 0 = no touch, coded as 1 = touch). We initiated our analysis with a model selection process, utilising the Akaike Information Criterion (AIC) as the primary criterion for model selection. This was complemented by the application of the ‘sw’ (Sum of Weights) function to extract measures of variable importance. This approach facilitated the identification of the ‘best model’, characterised by the lowest AIC value and sum of weights for each variable exceeding 0.7. Fixed factors were *Group* (Crozet Islands, Kerguelen Islands), *Order* (sequence of novel objects presented), and *Object* (yellow buoy, black plastic bag, orange wood plank) along with their interactions with *Group*. To account for breeding pair variability, we set pair identity as a random variable. In instances where the best model was effectively a null model—indicating that no fixed variables were selected—we concluded that none of the variables exerted a significant effect on the response variable, thereby halting further model consideration.

To assess whether *approach* (i.e. prediction 2; the time elapsed in seconds from the start of the trial to when the bird came within one body length of the novel object) and *touch* latencies (i.e. prediction 4; the time elapsed in seconds from the start of the trial to when the bird used its beak to contact the novel object) significantly differed between the groups, we applied survival analysis techniques. Consistent with the methods used for *predictions 1* and *3*, we followed the same model selection process and incorporated identical fixed factors (i.e. *Group*, *Order*, and *Object*). Specifically, we implemented Cox proportional hazards models with mixed effects (e.g. Sol et al. 2011). This method is particularly well suited for handling censored data which, in our study, occurred when a bird did not approach within the 360-s trial duration. The mixed effects component of the model allowed us to accommodate with the non-independence of observations.

Statistical analyses were performed using R version 3.6 (R Core Team 2019) and we used the packages *vif* for checking collinearity (Fox and Weisberg 2011), *MuMIn* for model selection and variable importance measures (i.e. ‘sw’ extractor function, Bartón 2020), *lme4* for performing GLMM analyses (Bates et al. 2015) and *coxme* for conducting mixed effects Cox models (Therneau 2022).

## Results

### Prediction 1: propensity to approach novel objects does not differ between groups

**Fig. 2:**
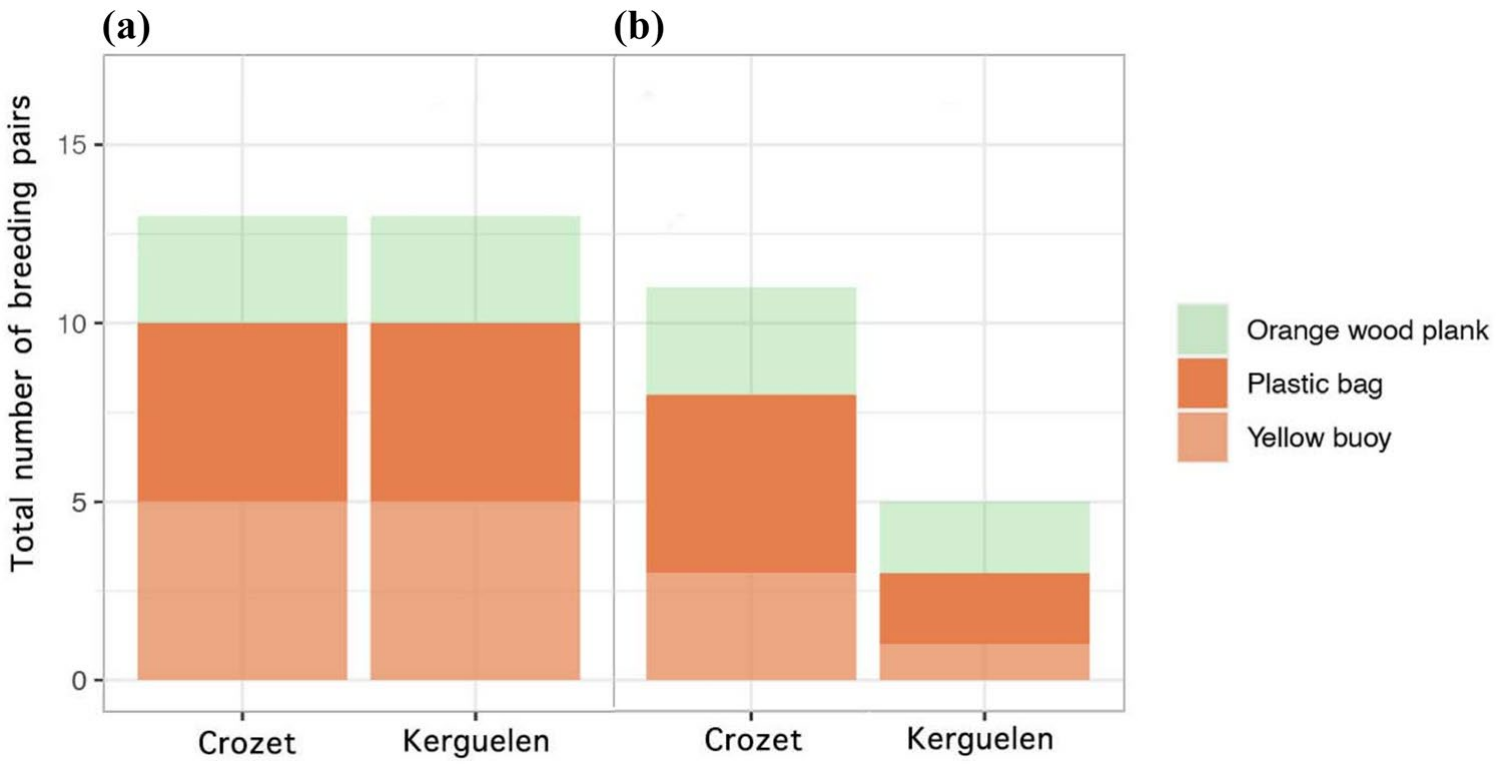
Stacked histograms showing the total number of **a** approach and **b** touch behaviours made by breeding pairs towards each novel object in the Crozet and Kerguelen groups

In both groups, at least one breeding partner in all pairs approached one of the three novel objects presented (Fig. 2). The best-fitting model (preliminary model comparisons based on the weakest Akaike’s criterion) was the null model (Table S1), which was supported by the extractor function (sum of weights were 0.39 for *Object,* 0.36 for *Order,* 0.35 for *Group,* 0.17 for Order*Group, and < 0.01 for Object*Group). Since no fixed variable(s) explained our response variable (i.e. propensity to approach), model selection was not further investigated.

### Prediction 2: latency to approach novel objects differs between groups

The best-fitting model (preliminary model comparisons based on the weakest Akaike’s criterion) was the null model (Table S2), which was supported by the extractor function (sum of weights were 0.33 for *Object,* 0.38 for *Order,* 0.36 for *Group,* 0.16 for Order*Group, and < 0.01 for Object*Group). Since no fixed variable(s) explained our response variable (i.e. propensity to approach), model selection was not further investigated.

### Prediction 3: propensity to touch novel objects differs between groups

At least one breeding partner in all pairs touched one of the three novel objects presented in the Crozet group, compared to three individuals in the Kerguelen group (Fig. 2). The best-fitting model (preliminary model comparisons based on the weakest Akaike’s criterion), which showed no correlation between our variables of interest (i.e. *Group, Object* and *Order*), was explained by *Group* (AICc = 52.1; Table S3). Model selection was supported by the extractor function (sum of weights were 0.76 for *Group*, 0.16 for *Object* and 0.43 for *Order,* 0.19 for Order*Group, and < 0.01 for Object*Group). We, therefore, assessed how exploration varied with *Group* and found that sheathbills from the Crozet group touched significantly more novel objects than birds from the Kerguelen group (binomial GLMM, z = −1.97, *p* = 0.04).

### Prediction 4: latency to touch novel objects differs between groups

The best-fitting model was explained by *Group* (AICc = 104.6; Table S4). Model selection was supported by the extractor function (sum of weights were 0.82 for *Group*, 0.26 for *Object,* 0.48 for *Order,* 0.21 for Order*Group and 0.02 for Object*Group). We found that sheathbills from the Crozet group were faster to touch novel objects than birds from the Kerguelen group (Cox model: z = −1.98, *p* = 0.04).

## Discussion

We examined responses to novelty in breeding pairs of sheathbills on two sub-Antarctic islands differing in environmental variability. Overall, all breeding pairs on both islands approached the novel objects, demonstrating low levels of neophobia (i.e. approaching the objects within a few minutes (Mettke‐Hofmann et al. 2002), similarly to other avian species tested in the field, e.g. urban caracaras, *Milvago chimango* (Biondi et al. 2020), skuas (Danel et al. 2022)). We also found no differences between the two populations in the latency to approach novel objects. However, the Crozet group touched significantly more and approached faster the novel objects compared to the Kerguelen group. Finally, contrary to other avian species (e.g. Biondi et al. 2015), stimulus typology (different objects) had no influence on sheathbills’ approach or touch behaviours, at least amongst the objects we used.

Caution is required since this group-specific difference in exploration has been investigated using two populations with a limited sample size (*n* = 6 breeding pairs). Furthermore, our findings may also have resulted from other—or a combination of—influencing factors. Indeed, since our two tested subspecies do not disperse at other islands (Burger 1979), one may argue that the difference between our two locations is not due to ecological variability. However, there is a level of heterozygosity three times higher in populations at Kerguelen Islands than Crozet Islands (Viot et al. 1993), making aspects such as habitat diversity potential influential factors (Jouventin et al. 1997, see also Bost et al. 1992; Danel et al. 2021). Moreover, interaction with humans and/or exposure to anthropogenic stimuli may promote exploration in some species (e.g. Donaldson et al. 2010; Damerius et al. 2017). In one of the few studies conducted on wild birds, Danel et al. (2022) raised the possibility that adult wild skuas, which had interacted with humans during food-rewarded behavioural and cognitive experiments, may have developed an increased tendency to explore novel objects presented by humans. Both species cohabit islands with humans and face similar histories of exposure to humans at certain locations, potentially giving rise to similar levels of motivation to gain information through interacting with novelty. Since 1964, sheathbills at Crozet have had many opportunities to interact with humans who visit daily the permanent research station inside the island’s king penguin colony—for example, sheathbills often try to steal objects or food from scientists’ belongings. Although further investigations are needed, such experiences may have reinforced sheathbills’ attraction to humans and exploration tendencies, through associative learning of the relationship between food and humans (Goumas et al. 2020).

Knowledge about the behaviour of insular endemic species, notably those with poor flying abilities (i.e. physically ‘trapped’ species, Olesen 2022), is fundamental to developing relevant applications for conservation. Rats (*Rattus rattus, Rattus norvegicus*) are the only introduced predator at Ile de la Possession in Crozet Islands, and they have had a negative impact on ecosystem (Pisanu et al. 2011). In some parts in the Indian Ocean’s subantarctic, eradication plans have been devised to wipe out introduced rats using ‘novel objects’ such as brightly coloured food-poisoned pellets. Since we now know that sheathbills tend to touch unfamiliar coloured items at Crozet Islands, future conservation measures of this kind will need to be managed with caution.

To conclude, high environmental variability, potentially combined with regular exposure to humans, are suggested explanations for the source of variation in response to novelty between our two test populations. In the near future, cognitive experiments involving regular seasonal food-related human–sheathbill interaction will be conducted at *île Verte*. This will provide exciting research opportunities for (i) comparing populations and/or subspecies that vary in their foraging habitat (e.g. sheathbills that live at locations where the intertidal zone is almost absent, or near penguin areas) and experience with humans, (ii) assessing the potential effect of object properties (e.g. by presenting ‘complex’, non-pre-existing novel objects e.g. Biondi et al. 2015; Miller et al. 2022) and sex (e.g. male sheathbills engage in more territorial aggression than females: Burger 1980, see also Shaw 1986) on exploration behaviour, and determining (iii) the cause-and-effect relationship between cognition and exploration, and (iv) to what extent exploration is flexible (e.g. adapted or learnt) and may develop after long-term human habituation.

## Supplementary Information

Below is the link to the electronic supplementary material.

Supplementary file1 (PDF 159 KB)

## Data Availability

Data supporting the findings of this study are available within Supplementary Information (provided in Table S3).
