# Supplementary material for: Responses to novelty in wild insular birds: comparing breeding populations in ecologically contrasting habitats
Source: Anim Cogn. 2024 Mar 2;27(1):4. doi: 10.1007/s10071-024-01838-w (PMC10907422; doi:10.1007/s10071-024-01838-w)
Supplement: Supplementary file 1 — Supplementary file1 (PDF 159 KB) [file 10071_2024_1838_MOESM1_ESM.pdf]

Supplementary Material

Responses to novelty in wild insular birds: comparing breeding populations in ecologically contrasting habitats

Samara Danel, Nancy Rebout, Léna Bureau, Timothée Zidat, Dora Biro, & Francesco Bonadonna

Results

Table S1 – Model selection table for *Prediction 1: Propensity to approach novel objects does not differ between groups*

	(Int)	Object	Order	Group	Obj:Gro	Ord:Gro	df	logLik	AICc	delta	weight
1	0.9586000	-	-	-	-	-	2	-21.270	46.9	0.00	0.281
2	-0.0004922	+	-	-	-	-	4	-19.094	47.5	0.57	0.211
23	2.9910000	-	-0.9344	+	-	+	5	-18.302	48.6	1.70	0.120

Table S2 – Model selection table for *Prediction 2: Latency to approach novel objects does not differ between groups*

	Object	Order	Group	Obj:Gro	Ord:Gro	df	logLik	AICc	delta	weight
1	-	-	-	-	-	0	-80.611	161.2	0.00	0.287
2	+	-	-	-	-	3	-76.432	162.1	0.88	0.185
23	-	-0.48990	+	-	+	3	-77.945	162.7	1.42	0.141

Table S3 – Model selection table for *Prediction 3: Propensity to touch novel objects differs between groups*

	(Int)	Object	Order	Group	Obj:Gro	Ord:Gro	df	logLik	AICc	delta	weight
5	4.520e-01	-	-	+	-	-	3	-22.664	52.1	0.00	0.341
23	2.904e+00	-	-1.1760	+	-	+	5	-20.790	53.6	1.50	0.161
1	-2.231e-01	-	-	-	-	-	2	-24.731	53.8	1.75	0.142

Table S4 – Model selection table for *Prediction 4: Latency to touch novel objects differs between groups*

	Object	Order	Group	Obj:Gro	Ord:Gro	df	logLik	AICc	delta	weight
5	-	-	+	-	-	1	-51.247	104.6	0.00	0.271
23	-	-0.7278	+	-	+	3	-49.347	105.5	0.83	0.179
7	-	-0.3406	+	-	-	2	-50.683	105.7	1.12	0.155

Table S5 – Raw data for approach and touch behaviours

Subspecies	Pair	Object	Order	Approach	Touch
Crozet	1	bag	1	1	1
Crozet	1	buoy	2	1	0
Crozet	1	plank	3	1	1
Crozet	2	bag	2	1	1
Crozet	2	buoy	1	1	1
Crozet	2	plank	3	0	0
Crozet	3	bag	3	1	1
Crozet	3	buoy	2	1	1
Crozet	3	plank	1	1	1
Crozet	4	bag	1	1	1
Crozet	4	buoy	3	1	0
Crozet	4	plank	2	0	0
Crozet	5	bag	2	0	0
Crozet	5	buoy	1	1	1
Crozet	5	plank	3	0	0
Crozet	6	bag	3	1	1
Crozet	6	buoy	2	0	0
Crozet	6	plank	1	1	1
Kerguelen	1	bag	2	1	0
Kerguelen	1	buoy	3	1	0
Kerguelen	1	plank	1	0	0
Kerguelen	2	bag	2	1	0
Kerguelen	2	buoy	1	1	0
Kerguelen	2	plank	3	1	0
Kerguelen	3	bag	2	1	1
Kerguelen	3	buoy	3	1	0
Kerguelen	3	plank	1	0	0
Kerguelen	4	bag	2	1	1
Kerguelen	4	buoy	3	1	1
Kerguelen	4	plank	1	1	1
Kerguelen	5	bag	2	0	0
Kerguelen	5	buoy	1	0	0
Kerguelen	5	plank	3	1	1
Kerguelen	6	bag	3	1	0
Kerguelen	6	buoy	1	1	0
Kerguelen	6	plank	2	0	0
